# Carbon Nanotube Mode-Locked Thulium Fiber Laser With 200 nm Tuning Range

**DOI:** 10.1038/srep45109

**Published:** 2017-03-21

**Authors:** Yafei Meng, Yao Li, Yongbing Xu, Fengqiu Wang

**Affiliations:** 1 School of Electronic Science and Engineering and Collaborative Innovation Center of Advanced Microstructures, Nanjing University, Nanjing, 210023, China

## Abstract

We demonstrated a mode-locked thulium/holmium (Tm/Ho) fiber laser continuously tunable across 200 nm (from 1860 nm to 2060 nm), which to the best of our knowledge represents the widest tuning range ever achieved for a passively mode-locked fiber laser oscillator. The combined use of a broadband carbon nanotube (CNT) saturable absorber and a diffraction grating mirror ensures ultra-broad tuning range, superb stability and repeatability, and makes the demonstrated laser a highly practical source for spectroscopy, imaging and optical communications. The laser emits <5 ps pulses with an optical spectral bandwidth of ∼3 nm across the full tuning range. Our results indicate that carbon nanotubes can be an excellent saturable absorber for achieving gain-bandwidth-limited tunable operation for 2 *μ*m thulium fiber lasers.

Mode-locked fiber lasers operating in the 2 *μ*m region have attracted considerable attention due to various applications in medical surgery, free space optical communications, light detection and ranging (LIDAR), nonlinear frequency conversion and transparent material processing^1^,^2^,^3^,^4^,^5^,^6^. Thulium and holmium ions based active fibers show a broad amplification bandwidth typically from 1.85–2.1 *μ*m and are therefore good candidates for ultrashort pulse generation and wideband wavelength tuning. For example, utilizing the full gain bandwidth (>200 nm) of thulium-doped fiber would allow for the generation of sub 30 fs pulses directly from a mode-locked oscillator^7^. Several techniques have been developed for generating ultrafast pulses including semiconductor saturable absorber mirrors (SESAMs)^8^, nonlinear polarization evolution (NPE)^9^,^10^ and nonlinear amplifying loop mirror (NALM)^11^. In recent years, low-dimensional carbon nanomaterials such as carbon nanotubes (CNTs)^12^,^13^,^14^ and graphene^15^,^16^ have been intensively investigated for ultrashort pulse generation at 2 *μ*m. This has further led to the investigations of other forms of novel materials such as topological insulators^17^,^18^, transition-metal dichalcogenides^19^,^20^ and black phosphorus^21^,^22^.

Despite significant progress for 2 *μ*m mode-locked lasers, most of the prior works focused on operation at a fixed wavelength which may limit their application potential in scenarios where wavelength tuning is desirable, such as frequency comb generation, molecular absorption line detection, time resolved nonlinear photon spectroscopy and optical parametric oscillation (OPO)^23^,^24^,^25^,^26^,^27^,^28^. Thus far, only a few works reported wavelength tunable operations. For example, through mechanically bending of a multimode interference filter (MMIF), a 95 nm tuning range is achieved for a mode-locked thulium fiber laser^29^. A tapered fiber device was used to modulate the peak insertion loss and combined with a CNT absorber, a wavelength tuning range of 50 nm is reported^30^. A graphene saturable absorber deposited on micro-fiber exhibits not only saturable absorption but also a strong polarization-dependent band-pass filtering effect. By controlling the intra-cavity polarization state, a wavelength tuning range of 60 nm is achieved^31^. Moreover, for a fiber laser mode-locked by NPE, it is demonstrated that the wavelength dependent insertion loss induced by the linear and nonlinear phase can be harnessed to achieve a 104 nm tuning range^32^. By utilizing a bidirectional pumping scheme, this is further optimized to show a 136 nm tuning range^33^.

To access the entire gain-bandwidth offered by thulium dopants (in excess of 200 nm) is of significant practical importance. Such system would provide a robust, low-cost and field-deployable source that are not currently being met by the more bulky solutions based on conventional OPOs. To this end, a broadband saturable absorber and wavelength tuning mechanism are required. Both CNTs and graphene are good candidates for broadband saturable absorbers. For carbon nanotubes, the mixing of different diameters and chiralities facilitates broadband operations with enhanced modulation depth^34^,^35^. Graphene possesses ultra-broadband nonlinear absorption. However, its modulation depth (in absolute terms) is rather low (<2.3%). While this may not be a big issue for solid-state lasers, e.g. graphene is recently used to mode-lock a Cr^2+^: ZnSe laser with ~300 nm tunability^36^, it may indeed lead to difficulty of self-started operation for fiber lasers.

In this letter, by combining a CNT-SA with a diffraction grating based mirror, we demonstrated a passively mode-locked Tm/Ho fiber laser with an ultra-broadband tuning range of 200 nm (from 1860 nm to 2060 nm). Such a wavelength versatile source would be instrumental in studying nonlinear optical phenomena, including supercontinuum generation and fiber based OPO system. In addition, due to the inherently simple geometry, the demonstrated laser exhibits excellent stability and repeatability, making it a highly practical source for field-deployable instrumentation for spectroscopy, imaging, and optical communications. Table 1 compares the results in this work with previous wavelength tunable mode-locked fiber lasers operating at 2 *μ*m.

The CNT-SA used in this work plays a crucial role for achieving wide-band mode-locking. To ensure operation in the 2 *μ*m band, we used commercially available arc-discharge singe-wall nanotubes (Carbon solutions Inc.). CNT-SA films with 20 *μ*m thickness are fabricated using the solution processing method described in ref. 37. The tube diameter distribution of ~1.3–1.6 nm is confirmed by Raman spectroscopy. The measured linear absorption spectrum of the CNT-SA film is shown in Fig. 1(a). It can be seen that the CNT-SA exhibits a relatively high absorption in the gain region of the Tm^3+^-doped fiber, which is helpful for maintaining good modulation depth. Nonlinear absorption of the CNT-SA is characterized using a mode-locked thulium fiber laser, operating at 1950 nm (NPI Lasers, Inc.). A typical nonlinear absorption curve for 1950 nm excitation is shown in Fig. 1(b). The modulation depth and the saturable intensity were determined to be 42.3% and 117 MW/cm^2^, respectively.

The schematic diagram of the experimental setup is shown in Fig. 2. The tunable mode-locked Tm/Ho fiber laser is constructed with a linear cavity similar to our previous work^38^. The pump source is provided by a laser diode (LD) operating at 1.56 *μ*m. The LD has an average output power of 10 mW and is subsequently amplified by a commercial erbium-doped fiber amplifier (EDFA). The maximum output power of the EDFA is 1 W. The amplified 1.56 *μ*m pump is injected into the cavity through a 1550/2000 nm wavelength-division multiplexer (WDM). We use 2 m Tm/Ho co-doped fiber (TH512, CorActive) as the gain media. The gain fiber has a core diameter of 9 *μ*m and NA of 0.16. The core absorption of the fiber is measured to be 13 dB/m at the pump wavelength of 1550 nm. Wavelength tuning is achieved by a filter consisting of a fiber collimator and free space diffraction grating. The bandwidth of this collimator-grating filter is measured to be 6.5 nm by an amplified spontaneous emission (ASE) source at 2 *μ*m. The collimator has a working distance of 50 mm and an output beam diameter of 0.45 mm. The collimator is AR-coated at 2 *μ*m in order to minimize the insertion loss. The line density of the grating used in the experiment is 600 l/mm. The grating works under a littrow structure to form a linear cavity. Although the grating efficiency is optimized at wavelength of 1.6 *μ*m, we infer that the efficiency is still about 90% around 2 *μ*m for s-polarized light, which is sufficient for wavelength dependent components. Ultrashort pulse mode-locking is started by the CNT-SA. The CNT-SA is sandwiched between two FC-APC connectors as the mode-locker. A 20/80 coated fiber ferrule is used as the output of the laser cavity. The total fiber length in the cavity was 5.5 m including 2.0 m Tm/Ho co-doped fiber, and 3.5 m SMF-28e fiber (note also there is 10 cm free space distance between the collimator and grating). The dispersion values of the Tm/Ho co-doped fiber and the SMF-28e fiber were about −56 ps^2^/km and −80 ps^2^/km respectively at a wavelength of 1.95 *μ*m. The net cavity dispersion is thus estimated to be −0.392 ps^2^.

At first, we set the grating at a fixed angle. Continuous wave mode-locking at 1960 nm was obtained when the pump power reaches 240 mW. The mode-locked pulse train is recorded with a high speed photodiode (EOT-5000) and a digital oscilloscope (Agilent DSO-X3052A). Figure 3(a) and (b) show the pulse trains under different time scale. The time interval between adjacent pulses is 54 ns which matches well with the fundamental repetition rate of 18.4 MHz. Moreover, in Fig. 3(b), the rather small pulse intensity fluctuation indicates that the mode-locking regime is stable. This is further confirmed by the RF spectrum of the signals shown in Fig. 3(c). The RF spectrum is recorded with a signal analyzer (R&S, FSV 30). The frequency span is 20 MHz and the resolution bandwidth is 10 kHz. It is shown that the fundamental repetition rate has a side-band suppression ratio of over 60 dB. Besides, the RF spectrum within the frequency span of 2 GHz in the inset further proves that the operation is free from pronounced intensity modulation or pulse breaking. In addition, we monitored the output power of the laser for 36 hours. The power deviation is found to be within 1% as shown in Fig. 3(d), suggesting excellent long-term stability.

Then, in order to change the center wavelength of the Tm/Ho fiber laser, the grating is finely tuned. Compared with other schemes, wavelength tuning by grating is easy to control and shows a good repeatability^33^. The tunable optical spectra is recorded with an optical spectrum analyzer (OSA, Yokogawa AQ6375). In Fig. 4(a), it is shown that the center wavelength can be continuously tuned from 1860 nm to 2060 nm, which corresponds to a large portion of the available gain bandwidth of the active fiber. In addition, FWHM of the optical spectra at different wavelengths are extracted and shown in Fig. 4(b). It is observed that the spectral bandwidth becomes slightly narrower towards the long wavelength side, most probably caused by the diminishing gain coefficient of thulium fiber. The output bandwidth is found to be ~3 nm, about half of the FWHM of the collimator-grating based filter, in good agreement with previous work with intra-cavity bandpass filters^39^. We notice that when our laser is tuned to the long-wavelength edge of the tuning range, quite pronounced ASE were observed, though toward the center part, ASE performance are quite good. Similar results were observed in other works on wavelength-tunable laser at 2 *μ*m^40^,^41^. Fortunately, there are a number of ways to combat ASE. For example, employing an extra-cavity bandpass tunable filter (a fiber Bragg grating or etalon) may help get rid of the out-of-band ASE noise. Another way to mitigate the ASE noise is by employing a longer span of active fiber so that reabsorption in the fiber would lead to enhanced gain at longer wavelength, which in turn would effectively suppress ASE.

In the meantime, the pulse duration is analyzed by a mid-infrared autocorrelator (APE, Pulsecheck MIR). As the autocorrelator incorporates a photodiode for signal detection, its efficiency is much lower as compared with a photomultiplier tube (PMT). Thus, the output signal (~1 mW) from the mode-locked oscillator is hard to be directly measured. This technical limitation makes it difficult to get temporal information on the oscillator output, and has been encountered by other researchers. To overcome it, a thulium doped fiber amplifier, or TDFA (NPI Lasers Inc.) is employed to boost the output power to tens of mW so that the signal could be measured by the autocorrelator. Figure 5(a–d) shows the autocorrelation traces at 1860 nm, 1910 nm, 1960 nm and 2010 nm. The corresponding retrieved pulse duration is 2.38 ps, 2.43 ps, 2.43 ps and 2.83 ps, respectively (assuming a sech^2^ profile). The typical time-bandwidth product time-bandwidth product (TBP) at 1960 nm is 0.567, larger than the transform limited value of 0.315. This Gaussian-like pulse shape is typically observed in fiber lasers incorporating of a bandpass filter. Other factors including the unavoidable nonlinear broadening during the amplification process in the TDFA and the dispersion in the long fiber pigtails may also cause the TBP to deviate from the theoretical value. It is found the pulse duration stays rather constant while the center wavelength is tuned. However, the pulse duration beyond 2010 nm can not be measured in the experiment due to limited gain of the TDFA beyond 2010 nm.

To give an idea of how mode-locking conditions are achieved across the tuning range, we recorded the pump thresholds for continuous wave mode-locking at different wavelength in Fig. 6. Within the range from 1900 nm to 1980 nm, the variation of the pump threshold is negligible. For wavelengths below 1900 nm, we saw only slight increase in the pump threshold. While for wavelength towards the longer side, i.e. greater than 2010 nm, the pump threshold increases dramatically. For 2060 nm, the pump threshold reaches 720 mW. Beyond 2060 nm, the mode-locking threshold is limited by the available output power from the pump source. This higher threshold is attributed to the diminishing gain of the active fiber at longer wavelength and the insertion loss caused by the WDM and passive fiber. Meanwhile, we measured the laser output powers under single pulse lasing just above the pump threshold. The result in Fig. 6 shows that the laser outputs almost constant power (~1 mW) within the entire tuning range. So we can deduce that the dynamics for mode-locking is mainly determined by the saturation properties of the CNT-SA. It also proved that the CNT-SA used in our experiment is indeed a good candidate for wideband tunable mode-locking at 2 *μ*m.

In conclusion, we have demonstrated an ultrafast Tm/Ho fiber laser with a center wavelength tunable from 1860 nm to 2060 nm. To the best of our knowledge, the 200 nm tuning range reported herein is the widest that has ever been achieved for a fiber based mode-locked oscillator. The strong and broadband nonlinear absorption offered by CNT saturable absorber combined with the inherent simplicity in wavelength selection by the diffraction grating mirror leads to robust and repeatable operation. The wavelength tuning capabilities of such a source has practical advantages in terms of power scaling and reduced footprint compared with conventional bulk OPO system. It is expected to enable more practical laser instrumentations for spectroscopy, imaging and optical communications.

## Additional Information

**How to cite this article:** Meng, Y. *et al*. Carbon Nanotube Mode-Locked Thulium Fiber Laser with 200 nm Tuning Range. *Sci. Rep.* **7**, 45109; doi: 10.1038/srep45109 (2017).

**Publisher's note:** Springer Nature remains neutral with regard to jurisdictional claims in published maps and institutional affiliations.

## Figures and Tables

**Figure 1:**
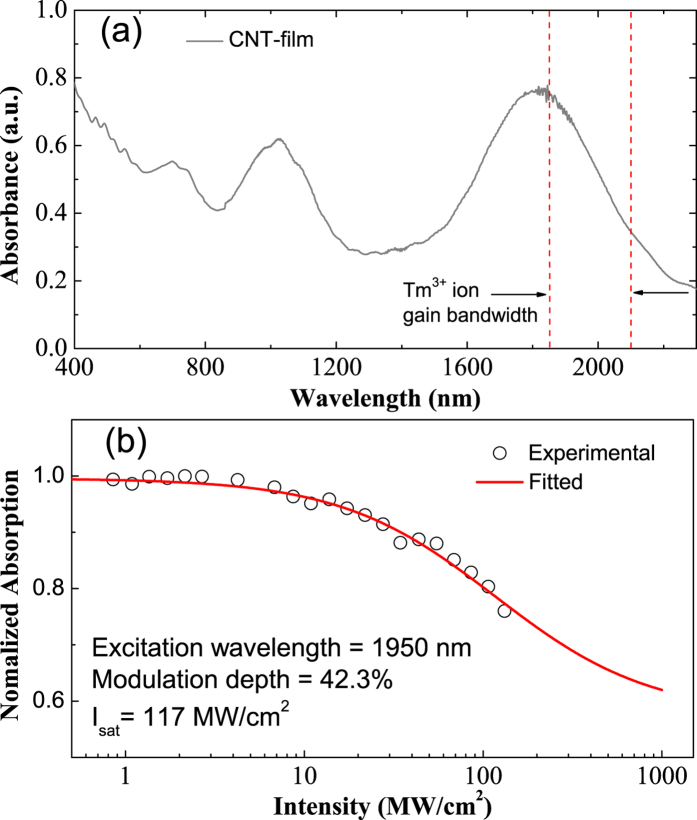
Optical characterization of the composite film. (**a**)Absorption spectrum of the CNT-SA film. The red dash lines mark the spectral gain region of Tm^3+^-doped fiber. (**b**) Normalized absorption of CNT-SA as a function of pump pulse peak intensity. The black bubbles are the experiment date and the red line is the fitting result.

**Figure 2:**
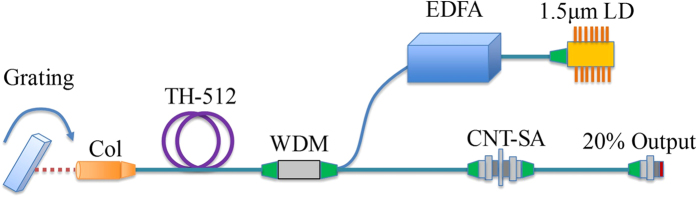
Schematic setup of the wavelength tunable mode-locked 2 *μ*m fiber laser. Col, collimator; WDM, wavelength-division multiplexer; EDFA, erbium doped fiber amplifier; CNT-SA, carbon nanotube-saturable absorber; LD, laser diode.

**Figure 3:**
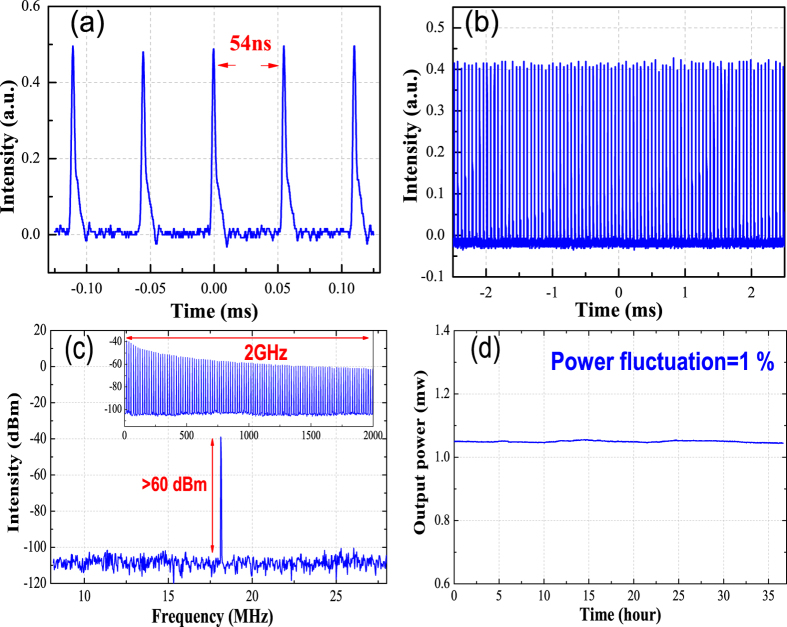
Mode-locked pulse trains. (**a**) At a time span of 0.3 ms and (**b**) at a time span of 5 ms, (**c**) RF spectrum of the mode-locked pulse trains and (**d**) Output power stability of mode-locked fiber laser.

**Figure 4:**
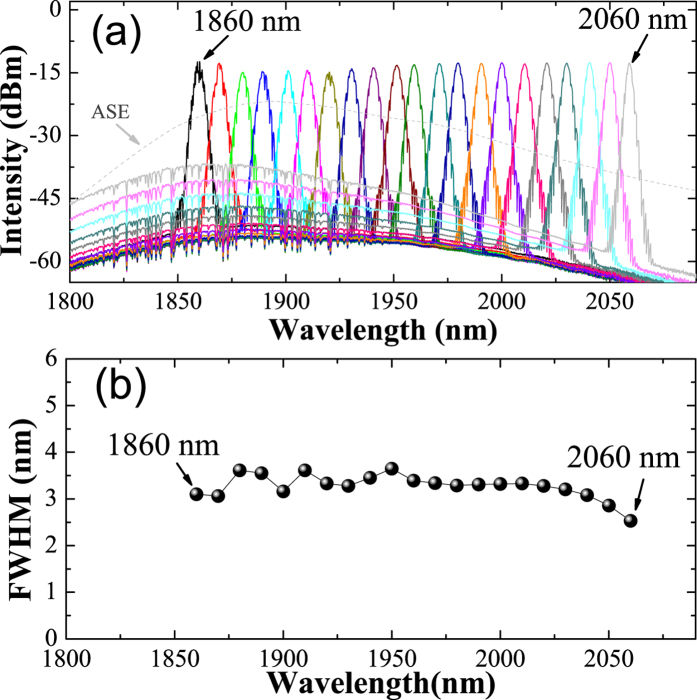
(**a**) Typical tunable mode-locked pulse spectrum measured with a wavelength interval of 10 nm. The dashed gray line is the ASE spectrum of the Tm/Ho fiber. (**b**) The FWHMs of the spectra at different wavelengths.

**Figure 5:**
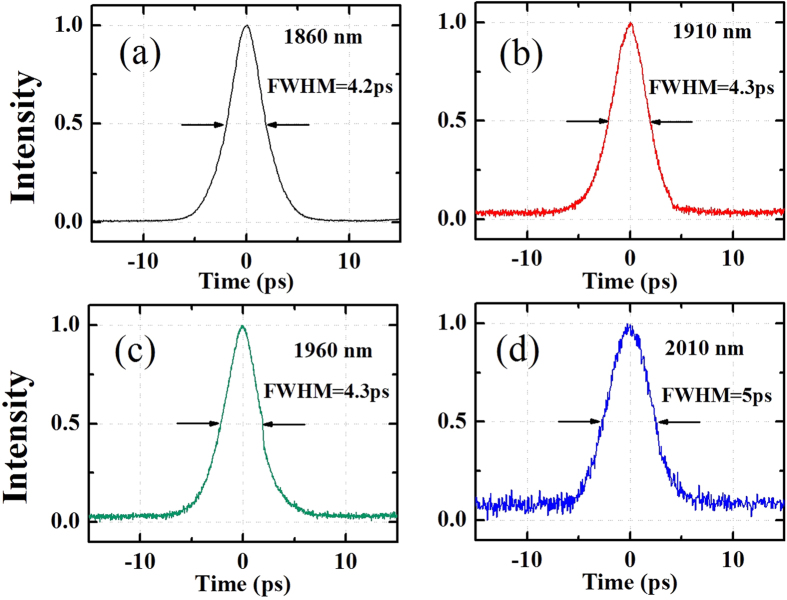
Autocorrelation traces measured with wavelength at (**a**) 1860 nm, (**b**) 1910 nm, (**c**) 1960 nm and (**d**) 2010 nm.

**Figure 6:**
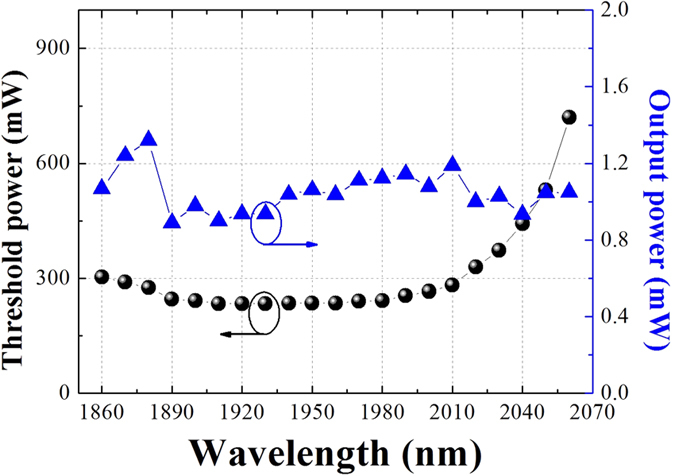
Pump threshold power and output power as a function of wavelength.

**Table 1:** Summary of broadly tunable mode-locked fiber lasers around 2 *μ*m.

Reference	Key method	Tuning Range	Year
Nelson *et al*.^32^	Filter based on NPE	1798–1902 nm (104 nm)	1995
Fang *et al*.^30^	Fiber-taper-filter & CNT absorber	1866.3–1916.4 nm (50.1 nm)	2010
Yang *et al*.^31^	Microfiber filter & graphene absorber	1880–1940 nm (60 nm)	2016
Li *et al*.^29^	Multimode interference filter (MMIT) & CNT absorber	1919.6–2014.9 nm (95.3 nm)	2016
Yan *et al*.^33^	Filter based on NPE & bidirectional pumping	1842–1978 nm (136 nm)	2016
***This work***	***Diffraction grating with littrow configuration & CNT- absorber***	***1860–2060 nm (200 nm)***	***2017***
